# Identifying the determinants of use of the G&G interventions for older adults in health and social care: protocol of a multilevel approach

**DOI:** 10.1186/s13104-015-1262-1

**Published:** 2015-07-07

**Authors:** Daphne Kuiper, Martine M Goedendorp, Robbert Sanderman, Sijmen A Reijneveld, Nardi Steverink

**Affiliations:** Department of Health Sciences, University Medical Center Groningen, University of Groningen, Groningen, The Netherlands; Department of Psychology, Health and Technology, University of Twente, Enschede, The Netherlands; Faculty of Behavioural and Social Sciences, University of Groningen, Groningen, The Netherlands

**Keywords:** Older adults, Self-management ability, Well-being, Loneliness, Evidence-based, Use, Implementation, Multilevel, Health, Social care

## Abstract

**Background:**

Despite aging-related losses, many older adults are able to maintain high levels of subjective well-being. However, not all older adults are able to self-manage and adapt. The GRIP&GLEAM [Dutch: GRIP&GLANS] (G&G) interventions have shown to significantly improve self-management ability, well-being and loneliness in older adults. Actual use of the evidence-based G&G interventions, however, remains limited as long as the interplay between implementation factors at different hierarchical stakeholder levels is poorly understood. The aim of the study is to identify the determinants of successful implementation of the G&G interventions.

**Methods/design:**

The study is performed in health and social care organizations in the northern part of the Netherlands. The degree of implementation success is operationalized by four parameters: use (yes/no), pace (time to initial use), performance (extent of use) and prolongation (intention to continue use). Based on the Fleuren model, factors at four hierarchical stakeholder levels (i.e. target group, professionals, organizations and financial-political context) are assessed at three measurement points in 2 years. The nested data are analyzed applying multilevel modeling techniques.

**Discussion:**

In this study, health and social care organizations are considered to be part of multilevel functional systems, in which factors at different hierarchical stakeholder levels impede or facilitate use of the G&G interventions. Strengths of the study are the multifaceted measurement of use, and the multilevel approach in identifying the determinants. The study will contribute to the development of ecologically valid implementation strategies of the G&G interventions and comparable evidence-based practices.

**Electronic supplementary material:**

The online version of this article (doi:10.1186/s13104-015-1262-1) contains supplementary material, which is available to authorized users.

## Background

Despite an increase in chronic diseases, people are living longer with less disability and fewer functional limitations [1]. Health, therefore, is recently being redefined into the more dynamic concept of ‘the ability to adapt and self-manage in the face of social, physical and emotional challenges’ [2]. Many older adults are able to adapt and self-manage, to maintain high levels of well-being, and to live independently up to very old age. Unfortunately, however, this does not hold for all older adults. Prevalence rates of loneliness [3, 4], social isolation [5], depression [6], and inactivity [7] in community-dwelling older adults are growing. Given the rapid increase in the number of older adults and the accumulation of the negative conditions mentioned, interventions that mitigate these conditions are called for [8].

The GRIP&GLEAM [Dutch: GRIP&GLANS] (G&G) interventions have shown to significantly improve self-management ability, well-being, and loneliness in older adults [9, 10]. Based on a common theoretical concept [11], two interventions have been developed: the G&G home visits and the G&G group course. Both interventions have been evaluated in randomized clinical trials [9, 10]. Positive effects were found on self-management ability, well-being and loneliness. The G&G interventions are designed for older people who have lost—or are at risk of losing—resources in several domains of functioning, which may lead to a diminished capacity for managing new losses or changes. Moreover, the G&G interventions are based on an explicitly positive concept: they focus on what individuals are still willing and able to do and not on the problems they are confronted with. The self-management abilities taught are not only intended as a response to loss but also as a tool to be used before loss has occurred. The G&G interventions are therefore also preventive in nature, aiming at the strengthening of one’s generative capacity to self-manage regarding all important aspects of well-being and health simultaneously [11].

Many older adults could benefit from the G&G interventions when the interventions would be routinely provided in health and social care services. However, the actual use of evidence-based practices (EBPs), such as the G&G interventions, remains limited in the Netherlands [12] as well as internationally [13, 14]. Despite the increasing availability of, and demand for, well-validated interventions, only about 50% of the interventions delivered in health care are evidence-based [15]. In social work this percentage appears to be even lower [16]. Moreover, actual use of EBPs is only significant to the extent that these practices are sustained for a longer period of time [17].

Three problems complicate the study of determinants of EBP-use. First, there is a wide array of facilitating and impeding factors affecting the use of EBPs. Systematic reviews produce comprehensive lists, ranging from 23 up to 50 different factors [18–22]. Second, EBP-use is a process, not an event [21]. Generally four stages are discerned: orientation, adoption, implementation (i.e. actual use) and continuation [19]. Empirical evidence which factor is important at what stage is, however, scarce. Third and last, different factors seem to operate on different hierarchical stakeholder levels, such as the individual level, the organizational, and the financial-political level [23]. This makes the study of determinants of EBP-use extra complex and explains why explicit multilevel studies on the determinants of EPB-use are also still scarce.

Historically, the focus of most studies has been on the individual level of the professional who is expected to change his routine in a way that enables the use of the new EBP [24]. Recently, a growing number of studies also encompass factors at the organizational and the financial-political level [25, 26]. A serious problem of these studies is, however, that the design and statistical methods are not fit to capture the complex interplay between phenomena at the several different hierarchical levels [15]. For example, self-efficacy (individual professional level), positive work climate (organizational level), and funding (financial-political context level) have been identified as important facilitating factors to EBP-use [23]. But, as of yet, it is not known which of these factors is decisive with respect to EBP-use in the presence of the other two. Answers to this type of questions can only be found when factors at more than one stakeholder level are assessed simultaneously, and when the nested data are analyzed employing multilevel modeling techniques.

The overall aim of the study is to identify the determinants of successful implementation of the G&G interventions. The concrete objectives of the study are, first, to describe the variation in actual use between organizations, expressed in terms of pace, performance and prolongation. And, second, to explain this variation at consecutive time points in the process by analyzing the factors at four hierarchical stakeholder levels (i.e. target group, professionals, organizations and financial-political context).

## Theoretical framework

In this study, the delineation of the stages of use, and the possible factors affecting the use of the G&G interventions, is theoretically informed by the model of Fleuren et al. [19]. In the past decade, a large number of models and frameworks on implementation processes has emerged [19, 20, 27–33]. Most of them acknowledge the multi-stage character (i.e. different consecutive phases) and the multi-level structure (i.e. factors at more than one stakeholder level) of the implementation process. We choose to use the Fleuren model above other models, because it incorporates the multi-level and multi-stage approach of implementation processes and combines it with comprehensiveness and practicality. No other model or framework seemed to give such detailed and clear directions to decide, for each stakeholder level, which factor could be important at what stage in the implementation process [34].

Categorized in four levels, the Fleuren model provides a list of 50 factors. A full description of each factor is given, as well as expectations about the direction of influence of each factor (e.g. ‘high staff turnover’ impedes and ‘low staff turnover’ facilitates implementation). This equipped us with adequate detail to prepare the content of the assessments in the consecutive measurement waves. For example, ‘formal reinforcement’ (a factor at the organizational level) is expected to facilitate the transition from adoption to initial implementation (i.e. the start of actual use) and ‘observability of effects’ (a factor at the professional level) is expected to facilitate the transition from implementation to continuation. Based on the Fleuren model, we were able to decide when to look at which factors and how to operationalize them.

**Figure 1:**
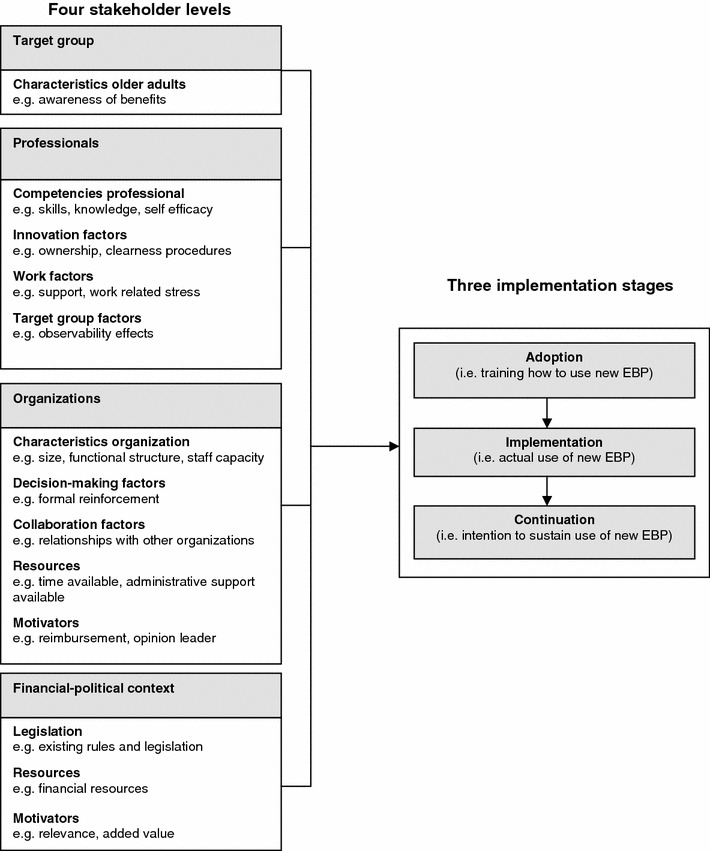
Theoretical framework.

Figure 1 shows the theoretical framework of the study. The process of innovation the organizations are expected to go through is divided into three stages: adoption, implementation and continuation. All possible factors of the Fleuren model are categorized at four stakeholder levels (i.e. target group, professionals, organizations and financial-political context). Moreover, within each stakeholder level, we sorted the factors into theoretically meaningful clusters, such as innovation-related factors, work-related factors, etc. In Additional file 1 the original model of Fleuren is described, as well as the minor adaptations we made.

## Methods

### Overview of the project

The study described in this protocol is part of a larger project that aims to promote and support the use of the evidence-based G&G interventions in health and social care organizations in the northern part of the Netherlands. Besides identifying the determinants of use of the G&G interventions, another goal of the larger project is to determine the effectiveness of the G&G interventions again. However, the study protocol at hand will only describe the former study, i.e. the study on the determinants of successful implementation of the G&G interventions.

Four partly overlapping phases can be distinguished in the study at hand. In phase 1 the G&G interventions are disseminated to the field of social and health care organizations. This is being done by means of G&G workshops, given by the G&G project team, at strategic meetings where professionals and managers of organizations gather. Because the larger project, of which this study is part, is not a ‘top-down’ initiative, organizations participate voluntarily. So, any organization that wants to adopt the G&G-interventions can take part in the project. The goal of phase 1 is to motivate at least 15 organizations to adopt the G&G interventions and participate in the study.

In phase 2 at least 30 professionals (two per organization) are trained to perform the G&G interventions. In phase 3 the trained professionals start implementing the G&G interventions in their organizations by recruiting older adults for participation and, subsequently, delivering the G&G interventions to them. The core of the empirical study takes place in phase 3. During that phase, the stages of implementation each organization goes through, are being monitored continuously by the project team, and the facilitating and impeding factors will be assessed at all stakeholder levels in three data collection waves. In phase 4 the data analyses will be executed. A detailed description of the four phases is given in Additional file 2.

The study protocol has been evaluated by the ethics committee of the University Medical Center of Groningen in May 2010. The study was considered to evaluate care as usual and therefore the study was exempted from the Medical Research Involving Human Subjects Act. The study was further performed in accordance with the Helsinki declaration. Informed consent will be given orally.

### The interventions

The two G&G interventions have the same theoretical basis [11], but are available in two delivery modes: the G&G home visits and the G&G group course. Both are considered in the empirical study at hand. The G&G home visits are delivered by a G&G coach in six individual home visits of 1.5 h. The G&G group course is delivered by two G&G teachers in 6 weekly meetings of 2.5 h and a booster session after 3 months. The home visits are intended to be delivered to both women and men, aged ≥65 years, who are physically and psycho-socially vulnerable and unable to travel to a group location. The group course is intended to be delivered to a group of around n = 10 socially vulnerable women, aged ≥55 years, who subscribe individually, and are physically capable of travelling to a group location. Both G&G interventions are described in detail in manuals, one for the G&G coach and one for the G&G teachers. There is also a workbook for each participant. The content of the G&G interventions is described in detail elsewhere [9, 10].

The training by which professionals become a certified G&G coach or G&G teacher involves two and a half days, and is given by master trainers of the G&G Program of the University Medical Center of the University of Groningen. In the first part of the training, the theoretical body of thought behind the G&G interventions is explained. In the second part, the intervention-manual is practiced through modeling and role-play. At the end of the training the professionals are being instructed on the content of the G&G implementation toolkit, which is developed by the G&G project team, and which offers a variety of materials supporting their implementation activities (e.g. PR materials, press release examples, brochures, etc.). The trained professionals are also informed about various facilitating activities offered by the G&G project team (i.e. website, annual work conference, and site visits).

### Study setting

The study is performed in health and social care organizations for older adults in the northern part of the Netherlands. Since 2007 municipal authorities are responsible for supervision and execution of the Social Support Act, which prescribes that vulnerable older adults and other vulnerable citizens need to be supported to recapture or maintain their ability to manage their own well-being. In consultation with the management of health and social care organizations, municipal policies are determined and available resources are allocated. Each municipality has one or more health and social care organizations that employ a variety of professionals. Professionals can either be social workers employed in welfare organizations or health professionals employed in home care organizations, providing both physical and psychosocial care to their clients. They can also be activity leaders employed in retirement homes, striving to empower residents and older adults living in sheltered accommodations next to the home.

### Study sample

**Figure 2:**
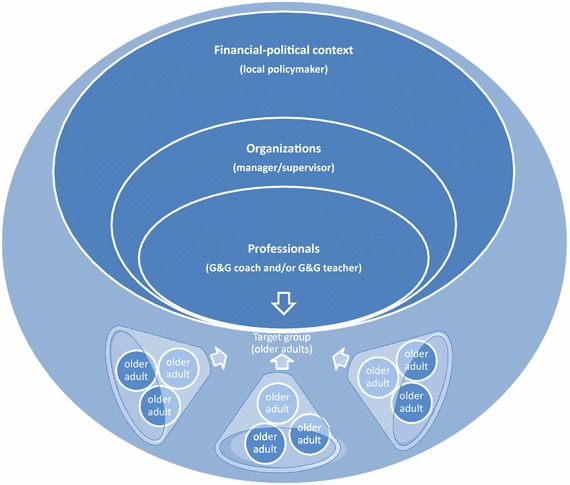
Hierarchical stakeholder levels.

The study sample consists of actors at four different hierarchical stakeholder levels (i.e. target group, professionals, organizations and financial-political context) as depicted in Figure 2. Therefore, there are four groups of informants.

The first group (i.e. the target group) consists of the older adults at risk of becoming vulnerable. The second group are the trained professionals, who deliver the G&G interventions. The third group consists of the managers of the participating organizations. When organizations have multiple management-layers, the manager who is closest to the work floor will be invited to act as key-informant. The fourth and final group consists of key informants at the level of the financial-political context. These are local policymakers who are well informed on the execution of the Social Support Act. They will be invited to act as informants for our study.

Based on experiences from an earlier pilot-implementation of the G&G interventions, it is feasible to include at least 15 new organizations in a period of 12 months [35]. Counting with 15 organizations, the sample size at the organizational and financial-political level will be 15 managers and 15 local policymakers. With a minimum of two G&G professionals per organization, the sample size at the professional level will be at least 30 G&G professionals.

With respect to the reach of the target group, concrete performance goals are communicated with the G&G professionals. Each G&G coach is expected to deliver the G&G home visits to at least three older adults (15 G&G coaches × 3 home-visits × 1 participant = 45 participants) and each pair of G&G teachers is expected to deliver at least three group courses, with an average of ten older adults per course (15 G&G teacher pairs × 3 group-courses × 10 participants = 450 participants). In the 2-year period of the data-collection, the sample size at the level of the target group will thus amount to approximately 495 older adults. Taking into account a drop-out rate of 8% [36] a maximum number of 400 older adults participating in the G&G interventions is expected to be feasible.

### Procedure and measures

The degree of implementation success will be assessed per organization, and is being operationalized by four parameters: use (yes/no), pace (time to initial use), performance (extent of use) and prolongation (intention to continue use beyond the timeframe of the study). The rationale behind the selection of these four parameters is that they assess the transitions between the three consecutive implementation stages in the Fleuren model (see Figure 1). The ‘use’ parameter measures the transition of organizations from adoption to implementation and the ‘prolongation’ parameter measures the transition from implementation to continuation. Next, we expect the ‘pace’ and ‘performance’ parameter to add to the explanation of both transitions.

Use, pace and performance can be easily assessed and with very high validity, because the actual performance of all organizations regarding the use of the G&G interventions will be monitored continuously throughout the study by the project team. The fourth and final parameter (i.e. intention to continue use of the G&G interventions beyond the time frame of the study) can necessarily only be measured as an estimation of the relevant actors. The intention of each professional, each manager, and each financial-political key informant, to continue the use of the G&G interventions beyond the timeframe of the study (i.e. prolongation) is operationalized with a single question with four answer categories ranging from (0) no intention to (3) strong intention. This question will be asked at the final measurement point of the study.

The facilitating and impeding factors that possibly affect the use of the G&G interventions are being measured at multiple measurement moments, simultaneously at the four hierarchical levels (i.e. target group, professionals, organizations and financial-political context). The content of the questionnaires varies somewhat per measurement point, because some factors only apply to the specific stage the organizations are in. For example, “ownership” is only applicable when users move from the adoption to the (initial) implementation stage, while “observability of effects” is applicable only when users move from the implementation to the continuation stage. In the following a brief outline of the measures is given. Details on the specific measurement moments, and at what point in time we measure which factors, are described in Additional File 3.

#### Target group

The impeding and facilitating factors at the level of the target group will be measured via the perception of the G&G professionals. This indirect way is necessary, because the factors at this level relate to possible participants, not to actual participants of the intervention. A sample of all possible participants (in the population) is hard to delineate. Therefore, the professionals will be asked to answer the questions of the target group level. The professionals will well be able to give an estimation of the impeding and facilitating factors that possibly play a role for older adults to participate (or not) in the G&G interventions, due to their large experience with the target group. The predefined factors of the theoretical framework at the level of the target group are thus translated into questions to be answered by the professionals. For example, the factor “awareness of benefits” is translated into the question: “Do you think older adults understand the benefits of participating in the G&G interventions?” The questions contain six answer categories ranging from (0) ‘not at all’ to (5) ‘completely’.

#### Professionals

The impeding and facilitating factors at the level of the professionals will be assessed by means of digital questionnaires. All professionals who have been trained and certified as G&G coach and/or G&G teacher will be invited to fill out the questionnaire. The predefined factors of the theoretical framework at the level of the professionals are translated into one or more questions per factor. For example, the factor “ownership” is translated into the question “To what extent do you feel responsible for G&G intervention start-up?” Each question contains six answer categories ranging from (0) ‘not at all’ to (5) ‘completely’.

#### Organizations

The impeding and facilitating factors at the level of the organizations will be measured by means of a telephone interview with the managers. The predefined factors of the theoretical framework at the level of the organization are translated into one or more questions per factor. For example, the factor “staff capacity” is translated into the question “Is your staff capacity sufficient to spend time on integrating the G&G interventions in routine practice?” The questions contain six answer categories ranging from (0) ‘not at all’ to (5) ‘completely’.

#### Financial-political context

The impeding and facilitating factors at the level of the financial-political context will also be measured by means of a telephone interview with a strategic or financial local policymaker. The predefined factors of the theoretical framework at this level are translated into one or more questions per factor. For example, the factor “added value” is translated into the question “To what extent do you think that the G&G interventions add something to the existing services for older people in your community?” The questions contain six answer categories ranging from (0) ‘not at all’ to (5) ‘completely’.

### Data analysis

All data will be imported in SPSS statistics 20. Descriptive analysis will be used to characterize use, time to initial use (pace), extent of use (performance) and the intention to continue use beyond the timeframe of the study (prolongation). Data collected at the four stakeholder levels will be merged and aggregated. Intra-class correlations will be calculated to assess the reliability of individual data aggregated at group levels in hierarchical models (i.e. professionals nested in organizations). The relevance of applying multilevel modeling to the data will be assessed by testing an unconditional or null model in which no predictors are specified. Only when significant variations in the dependent variables are present across organizations or municipalities, multilevel regression modeling will be applied. If no significant variation in use, pace, performance or prolongation is found across organizations or municipalities, we confine to single-level modeling techniques.

## Discussion

The aim of the study described in this protocol is to identify the determinants of successful implementation of the G&G interventions. In this study, health and social care organizations are considered to be part of multilevel functional systems [37], in which factors at different hierarchical levels can impede or facilitate the actual use of the G&G interventions. By assessing the unique contribution of target group, professional, organizational and financial-political context factors, as well as the complex interplay between these factors, results are expected to be of added value to the current scientific knowledge on barriers and facilitators to EBP-use.

The study has several strengths. The first is that implementation success in this study is not only assessed by the parameter use (yes/no), but it is also specified in three other indicators of use, namely: pace, performance and prolongation. This approach yields a much more specified insight in the various aspects of implementation success. Second, the possible factors that are considered in this study are theoretically supported by the Fleuren model, which provides a solid basis and prevents an ad hoc selection of possible factors. Finally, the analyses of the complex interplay between factors at different hierarchical stakeholder levels are executed with advanced multilevel modeling techniques. This is the optimal way of doing justice to the multi-layered nature of reality in implementation processes.

In conclusion, globally [38] and nationally [39] there is momentum to invest in EBPs that support aging individuals to live full, enriching and productive lives for as long and as much as possible. The G&G interventions are an example of such EBPs. They have been designed and tested in the last decade. Now it is time to increase our understanding of how to transport them to health and social care settings in a sustainable way. Identifying the determinants of successful implementation of the G&G interventions will also contribute to the development of ecologically valid implementation strategies of the G&G interventions and comparable new evidence-based practices.

## Additional files

Additional file 1: Two-step procedure constructing theoretical framework. Additional file 2: Time plan G&G-implementation study. Additional file 3: Factors, time points and number of items per stakeholder level.

## Abbreviations

G&G: GRIP&GLEAM [Dutch: GRIP&GLANS]; GRIP stands for the ability to adapt and self-manage. GLEAM stands for well-being and the feeling that life is good and worth living
EBP: Evidence Based Practice; a practice that has been established as effective through scientific research according to a set of explicit criteria
