# Supplementary material for: Identifying the determinants of use of the G&G interventions for older adults in health and social care: protocol of a multilevel approach
Source: BMC Res Notes. 2015 Jul 7;8:296. doi: 10.1186/s13104-015-1262-1 (PMC4493806; doi:10.1186/s13104-015-1262-1)
Supplement: Additional file 1: — Two-step procedure constructing theoretical framework. [file 13104_2015_1262_MOESM1_ESM.pdf]

Additional File 1

Title: Two-step procedure constructing theoretical framework

Although basically useful, we made some specific choices and adaptations regarding the Fleuren model as depicted below in Figure 1.

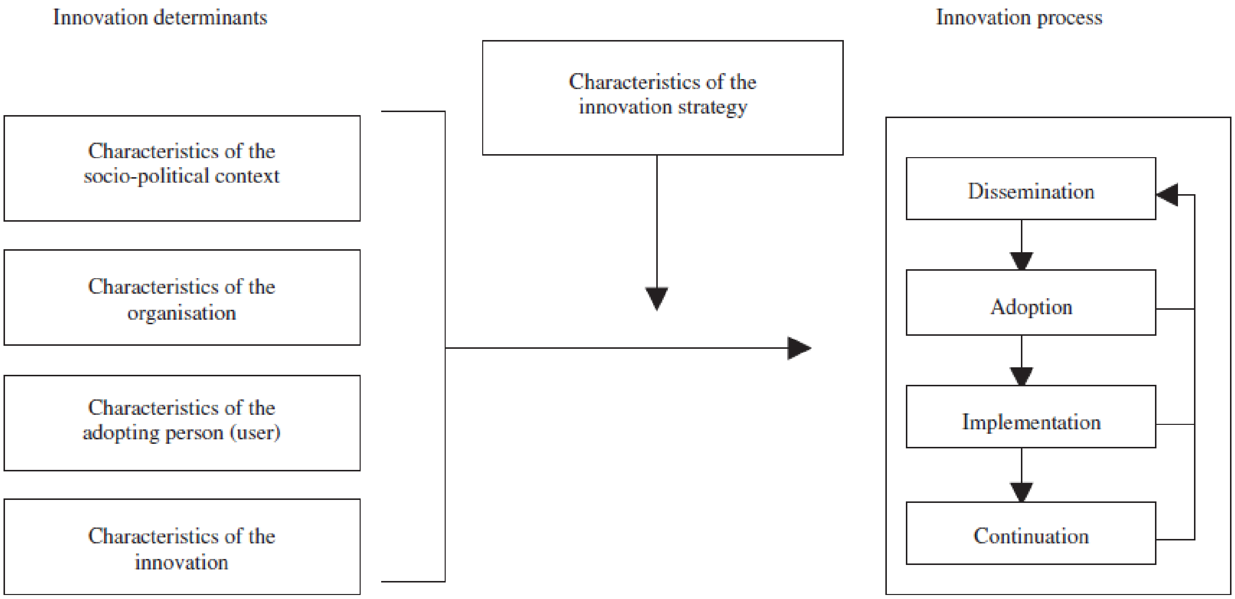

Figure 1 Framework representing the innovation process and related categories of determinants.

(From: Fleuren M, Wiefferink K, Paulussen T: Determinants of innovation within health care organizations: literature review and Delphi study. Int J Qual Health Care 2004, 16(2): p. 108, by permission of Oxford University Press).

First, in our study we concentrate on assessing the facilitating and impeding factors associated with especially the three last stages of the innovation process (i.e., the adoption, implementation, and continuation stage), not on the first dissemination stage.

Second, we chose to reorganize the levels and predefined factors somewhat as to make them more suitable to our study aims. Table 1 on page 3 of this document shows the two-step procedure that was followed to tailor the framework of Fleuren et al. to our purpose.

Step 1 consisted of adapting the four levels and the factors, because some levels and some factors are less applicable to our purpose. As a first adaptation, we decided to make a separate (new) level of the target group, because several of the factors on the level of the socio-political context [factors 1 to 5] would better fit to the level of the target group (i.e. the older adults) in our study. We believe that also at the level of the target group impeding and facilitating factors to successful implementation should be identified.

As a second adaptation, we left the level of the innovation as a separate level out, because, in our study, most of the factors on this level better fit to the level of the professionals delivering the G&G-interventions. For example, clearness of procedures [factor 34] or appealing to use [factor 39] relate to the intervention as it is being used by the professional.

The result of step 1 is shown in the middle column of Table 1, in which the four stakeholder levels are shown, and the predefined factors assigned to these four levels. Note that four factors [28, 29, 30 and 50] have been assigned to two levels, because these factors appear to apply to both.

In step 2 we sorted the separate factors within each level into meaningful clusters, because, in our opinion, several of the predefined factors of Fleuren et al. interrelate more closely than others. For example, skills [factor 24], knowledge [factor 25] and self-efficacy [factor 26] can be clustered as competencies of the professional, whereas reimbursement [factor 44] and opinion leader [factor 50] can be clustered as motivators at the level of the organizations. Clustering of factors adds to a more systematic classification, and subsequently helps in the process of operationalizing the factors into groups of items. Moreover, the term ‘patient’ is replaced by the term ‘client’, which is more applicable to the social care service setting of the study.

Table 1

STEP 1 → STEP 2 →

Original categorization Fleuren et al., 2004

Determinants related to socio-political context
[1] willingness patient to cooperate<br>[2] patient awareness of health benefits<br>[3] patient doubts expertise professional<br>[4] financial burden on patient<br>[5] patient discomfort<br>[6] existing rules, regulations and legislation

Determinants related to the organization
[7] decisionmaking process and procedures<br>[8] hierarchical structure<br>[9] formal reinforcement<br>[10] organizational size<br>[11] functional structure<br>[12] relationship with other organizations<br>[13] nature of collaboration internally<br>[14] staff turn-over<br>[15] staff capacity<br>[16] available expertise<br>[17] logistical procedures<br>[18] number of potential users<br>[43] financial resources<br>[44] reimbursement<br>[45] other resources available<br>[46] administrative support available<br>[47] time available<br>[48] availability of staff for coordination<br>[49] professionals involved in development<br>[50] opinion leader

Determinants related to the user
[19] support from colleagues<br>[20] support from other professionals<br>[21] support from supervisor<br>[22] support from higher management<br>[23] modeling<br>[24] skills<br>[25] knowledge<br>[26] self-efficacy<br>[27] ownership<br>[28] innovation - task orientation fit<br>[29] expectations cooperation target group<br>[30] expectations satisfaction target group<br>[31] work-related stress<br>[32] contradictive goals<br>[33] ethical problems

Determinants related to the innovation
[34] clearness procedures<br>[35] compatibility<br>[36] trialability<br>[37] relative advantage<br>[38] observability<br>[39] appealing to use<br>[40] relevance/added value<br>[41] risks to the patient<br>[42] frequency of use

New assignment to stakeholder levels

Target group
[1] willingness patient to cooperate<br>[2] patient awareness of health benefits<br>[3] patient doubts expertise professional<br>[4] financial burden on patient<br>[5] patient discomfort

Professionals
[19] support from colleagues<br>[20] support from other professionals<br>[21] support from supervisor<br>[22] support from higher management<br>[23] modeling<br>[24] skills<br>[25] knowledge<br>[26] self-efficacy<br>[27] ownership<br>[28] innovation - task orientation fit<br>[29] expectations cooperation target group<br>[30] expectations satisfaction target group<br>[31] work-related stress<br>[32] contradictive goals<br>[33] ethical problems<br>[34] clearness procedures<br>[36] trialability<br>[37] relative advantage<br>[38] observability<br>[39] appealing to use<br>[41] risks to the patient<br>[42] frequency of use<br>[49] professionals involved in development

Organizations
[7] decisionmaking process and procedures<br>[8] hierarchical structure<br>[9] formal reinforcement<br>[10] organizational size<br>[11] functional structure<br>[12] relationship with other organizations<br>[13] nature of collaboration internally<br>[14] staff turn-over<br>[15] staff capacity<br>[16] available expertise<br>[17] logistical procedures<br>[18] number of potential users<br>[28] innovation - task orientation fit<br>[29] expectations cooperation target group<br>[30] expectations satisfaction target group<br>[35] compatibility<br>[44] reimbursement<br>[45] other resources available<br>[46] administrative support available<br>[47] time available<br>[48] availability of staff for coordination<br>[50] opinion leader

Financial-political context
[6] existing rules, regulations and legislation<br>[43] financial resources<br>[40] relevance/added value<br>[50] opinion leader

Clustering subgroups within stakeholder levels

Stakeholder level	Subgroups
Target group	**Characteristics older adults**<br>[1] willingness client to cooperate<br>[2] client awareness of health benefits<br>[3] client doubts expertise professional<br>[4] financial burden on client<br>[5] client discomfort
Professionals	**Competencies professional**<br>[24] skills<br>[25] knowledge<br>[26] self-efficacy<br><br>**Innovation factors**<br>[27] ownership<br>[34] clearness procedures<br>[36] trialability<br>[37] relative advantage<br>[39] appealing to use<br>[42] frequency of use<br>[49] professionals involved in development<br><br>**Work factors**<br>[19] support from colleagues<br>[20] support from other professionals<br>[21] support from supervisor<br>[22] support from higher management<br>[23] modeling<br>[28] innovation - task orientation fit<br>[35] compatibility<br>[31] work-related stress<br>[32] contradictive goals<br><br>**Target group factors**<br>[29] expectations cooperation target group<br>[30] expectations satisfaction target group<br>[33] ethical problems<br>[38] observability<br>[41] risks to the client
Organizations	**Characteristics organization**<br>[10] organizational size<br>[11] functional structure<br>[14] staff turn-over<br>[15] staff capacity<br>[18] number of potential users<br>[28] innovation - task orientation fit<br>[29] expectations cooperation target group<br>[30] expectations satisfaction target group<br><br>**Decision-making factors**<br>[7] decisionmaking process and procedures<br>[8] hierarchical structure<br>[9] formal reinforcement<br><br>**Collaboration factors**<br>[12] relationship with other organizations<br>[13] nature of collaboration internally<br><br>**Resources**<br>[16] available expertise<br>[17] logistical procedures<br>[45] other resources available<br>[46] administrative support available<br>[47] time available<br>[48] availability of staff for coordination<br><br>**Motivators**<br>[44] reimbursement<br>[50] opinion leader
Fin.pol. context	**Legislation**<br>[6] existing rules, regulations and legislation<br><br>**Resources**<br>[43] financial resources<br><br>**Motivators**<br>[40] relevance/added value<br>[50] opinion leader
