# Supplementary material for: Identifying the determinants of use of the G&G interventions for older adults in health and social care: protocol of a multilevel approach
Source: BMC Res Notes. 2015 Jul 7;8:296. doi: 10.1186/s13104-015-1262-1 (PMC4493806; doi:10.1186/s13104-015-1262-1)
Supplement: Additional file 2: — Time plan G&G-implementation study. [file 13104_2015_1262_MOESM2_ESM.pdf]

Additional File 2

Title: Time plan G&G-implementation study

The G&G-implementation study is framed in time for 42 months, consisting of four overlapping phases. Main tasks and actions of both the health- and social care organizations and the G&G-project team are depicted below in the time plan and subsequently described in detail.

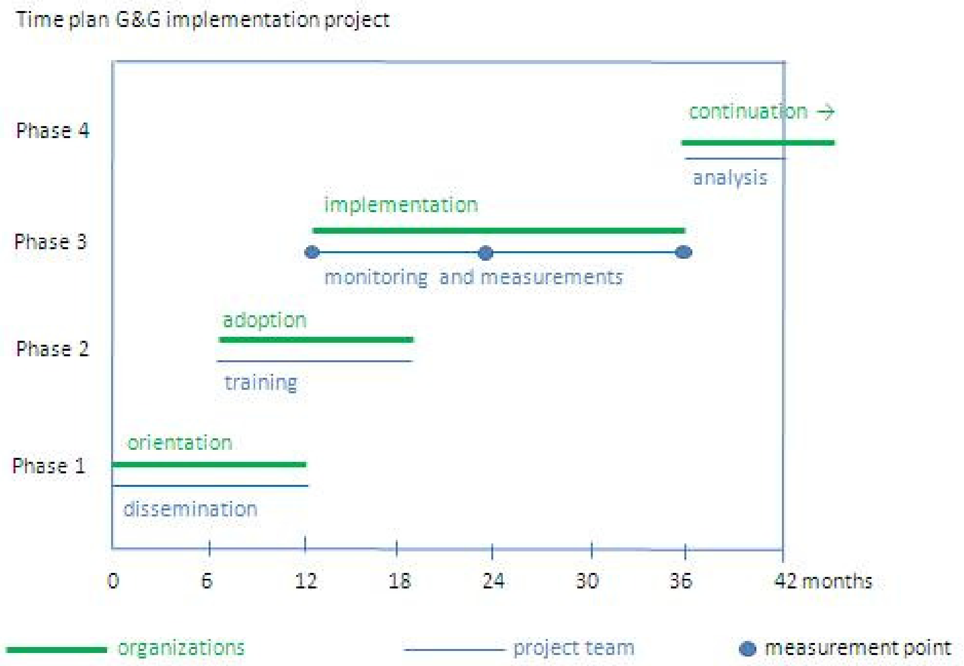

Phase 1

In phase 1 the innovative body of thought of the G&G-interventions is disseminated by the project team to the practice field of (vulnerable) older adults. Workshops demonstrated to be a feasible and effective dissemination strategy when piloting the G&G-interventions. Professionals, managers and/or local policymakers attending the workshops and 'tasting' a part of the intervention, have proven to be easily persuaded of the potential value of the G&G-interventions for vulnerable older adults. Professionals, managers and/or local policymakers considering adoption of the G&G-interventions, are informed by the G&G-project team on the prerogatives (e.g. the subsidized fee for training professionals) and the obligations (e.g. participation in monitoring and

assessment activities is mandatory) of becoming an implementation site in the study under consideration. The dissemination strategy will be deployed until saturation of the planned number of organizations (at least n=15) has been reached.

Phase 2

After having reached agreement on study participation a minimum number of two professionals per implementation site subscribe to the next G&G-coach or G&G-teacher-training. The training is executed by master trainers of the G&G-program of the UMCG by means of a structured training manual. The maximum number of professionals participating in the training amounts to n=12. The duration of both the G&G-coach and G&G-teacher training is two and a half consecutive days. Knowledge transfer on the SMW-theory (Steverink et al, 2005) and the educational tools to translate it into appealing and comprehensible language for older adults are central to the first training day. Skills training in instructional and modeling behavior are central to the second training day. Information on the (new developed) implementation toolkit and the way in which trained and certified professionals can ask for implementation support from the G&G-project team are communicated during the last training day. Moreover, a performance goal for the delivery of the G&G-interventions is communicated with the professionals in training.

Phase 3

Trained professionals can start implementing the G&G-interventions in their own organization immediately after being certified as coach and/or teacher by the G&G-program of the UMCG. Tools needed for recruitment of older adults (e.g. text for flyers or newspaper), intake and delivery of the G&G-interventions (e.g. PDF workbook for participants) are available upon request from the G&G-project team. Other ways to facilitate implementation efforts of the G&G professionals are the launch of the GRIP&GLANS-website [www.gripenglans.nl], an annual work conference and a site visit on request. All the above mentioned facilitating activities of the G&G-project team are voluntary and attended or given only on request of the adopting organization or professional.

Besides ongoing facilitation and monitoring, in phase 3 the assessment of the predefined factors from the Fleuren model (Fleuren et al, 2004) are performed by the G&G-project team. Participation in the measurements is introduced as mandatory to participating organizations and professionals.

Phase 4

In phase 4 the data analysis and reporting of results will be executed. Requests for support of implementation sites who continue to use the G&G-interventions, will be complied with.
