# Supplementary material for: Identifying the determinants of use of the G&G interventions for older adults in health and social care: protocol of a multilevel approach
Source: BMC Res Notes. 2015 Jul 7;8:296. doi: 10.1186/s13104-015-1262-1 (PMC4493806; doi:10.1186/s13104-015-1262-1)
Supplement: Additional file 3: — Factors, time points and number of items per stakeholder level. [file 13104_2015_1262_MOESM3_ESM.pdf]

Additional File 3

Title: factors[1], time points and number of items per stakeholder level

**Level target group[2]**		Number of items T1	Number of items T2	Number of items T3
**Characteristics older adults**				
[1]	willingness client to cooperate	-	1	1
[2]	clients awareness of benefits	-	1	1
[3]	clients doubts expertise professional	-	1	1
[4]	financial burden on client	-	1	1
[5]	client discomfort	-	2	2

**Level professionals**		Number of items T1	Number of items T2	Number of items T3
**Competencies professional**				
[24]	skills	5	-	-
[25]	knowledge	2	-	-
[26]	self-efficacy	4	4	4
**Innovation factors**				
[27]	ownership	3	2	2
[34]	clearness procedures	-	1	1
[36]	trial ability	na[a]	na[a]	na[a]
[37]	relative advantage	2	2	2
[39]	appealing to use	4	5	5
[42]	frequency of use	na[b]	na[b]	na[b]
[49]	professionals involved in development	na[a]	na[a]	na[a]
**Work factors**				
[19]	support from colleagues	2	2	2
[20]	support from other professionals	2	2	2
[21]	support from supervisor	2	2	2
[22]	support from higher management	2	2	2
[23]	modeling	2	2	2
[28]	innovation – task orientation fit	2	2	2
[35]	compatibility	2	2	2
[31]	work-related stress	8	8	8
[32]	contradictive goals	1	1	1
**Target group factors**				
[29]	expectations cooperation targetgroup	1	-	-
[30]	expectations satisfaction targetgroup	2	-	-

[1] Detailed descriptions of each factor can be found on page 110 and 111 in Fleuren M, Wiefferink K, Paulussen T: Determinants of innovation within health care organizations: literature review and Delphi study. *Int J Qual Health Care* 2004, 16(2):107-123

[2] The impeding and facilitating factors at the level of the target group will be measured indirectly, i.e. via the perception of the professionals.

[33]	ethical problems	na[c]	na[c]	na[c]
[38]	observabilty effects	-	1	1
[41]	risks to the client	na[c]	na[c]	na[c]

na = not applicable

na[a] = not applicable, because these factors were taken into consideration when the G&G-interventions were developed and tested. During the timeframe of this study, manuals and procedures for delivering the G&G-interventions are fixed.

na[b]= not applicable, because it interacts with a parameter of implementation success , i.e. performance (extent of use).

na[c]= not applicable, because participation in the G&G-interventions does not involve any ethical dilemmas for the professional or risks for the older adult

**Level organizations**		Number of items T1	Number of items T2	Number of items T3
**Characteristics organization**				
[10]	organizational size	-	4	4
[11]	functional structure	-	1	1
[14]	staff turn-over	-	1	1
[15]	staff capacity	-	1	1
[18]	number of potential users	-	fixed[3]	fixed
[27]	ownership	-	1	1
[28]	innovation – task orientation fit	-	2	2
[29]	expectations cooperation targetgroup	-	1	1
[30]	expectations satisfaction targetgroup	-	2	2
[35]	compatibility	-	1	1
**Decision-making factors**				
[7]	decision making process and procedures	-	1	1
[8]	hierarchical structure	-	1	1
[9]	formal reinforcement	-	2	2
**Collaboration factors**				
[12]	relationship with other organizations	-	3	3
[13]	nature of collaboration internally	-	1	1
**Recources**				
[16]	available expertise	-	1	1
[17]	logistical procedures	-	1	1
[45]	other resources available	-	1	1
[46]	administrative support available	-	1	1
[47]	time available	-	4	4
[48]	availability of staff for coordination	-	1	1
**Motivators**				
[44]	reimbursement	-	4	4
[50]	opinionleader	-	1	1

[3] Training of professionals to be a G&G coach and/or a G&G teacher is confined to month 6-18 of the project. So the number of potential users is known and fixed after month 18.

Level financial-political context		Number of items T1	Number of items T2	Number of items T3
**Legislation**				
[6]	existing rules, regulations and legislation	-	4	4
**Resources**				
[43]	financial resources	-	3	3
**Motivators**				
[40]	relevance/added value	-	2	2
[50]	opinionleader	-	3	3
